# PHD finger protein 5A promoted lung adenocarcinoma progression via alternative splicing

**DOI:** 10.1002/cam4.2115

**Published:** 2019-04-01

**Authors:** Shuangshuang Mao, Yuan Li, Zhiliang Lu, Yun Che, Jianbing Huang, Yuanyuan Lei, Yalong Wang, Chengming Liu, Xinfeng Wang, Sufei Zheng, Nan Sun, Jie He

**Affiliations:** ^1^ Department of Thoracic Surgery National Cancer Center/National Clinical Research Center for Cancer/Cancer Hospital, Chinese Academy of Medical Sciences and Peking Union Medical College Beijing China

**Keywords:** alternative splicing, lung adenocarcinoma, PHF5A, splicing factor

## Abstract

Alternative splicing (AS) and the regulation of AS by splicing factors play critical roles in cancer. Plant homeodomain (PHD)–finger domain protein PHF5A, a critical splicing factor involved in AS, has been demonstrated to play an oncogenic role in glioblastoma multiforme and breast cancer, but its biological function in lung cancer remains unclear. In the present study, we systematically analyzed the biological function and clinical relevance of PHF5A in non–small cell lung cancer (NSCLC). We found that PHF5A was significantly upregulated in NSCLC tumors compared with normal tissues in both TCGA data set and tissue microarrays. Upregulation of PHF5A was negatively correlated to the overall survival (OS) of lung adenocarcinoma (LUAD) patients. Loss‐of‐function and gain‐of‐function experiments confirmed that PHF5A functioned as an oncoprotein by promoting LUAD cell proliferation, migration and invasion, inducing G0/G1 cell cycle progression and inhibiting cisplatin–induced apoptosis. RNA–seq analysis identified many essential genes whose AS was dysregulated by PHF5A, including cell cycle–associated genes such as SKP2, CHEK2, ATR and apoptosis–associated genes such as API5 and BCL2L13. Additionally, pladienolide, a small molecular inhibitor of PHF5A, inhibited LUAD cell proliferation in a dose–dependent manner and induced AS changes similar to PHF5A knockdown. In conclusion, we validated that PHF5A played an oncogenic role via AS in LUAD and suggested that PHF5A might serve as a potential drug target with a promising anticancer therapeutic effect.

## 1 INTRODUCTION

Lung cancer has been the most common incident cancer and the leading cause of cancer death in China^1^ and worldwide, with an estimated 1.8 million new cases and 1.59 million deaths globally in 2012.^2^ Non–small cell lung cancer (NSCLC) is the major histological subtype of lung cancer accounting for approximately 85% of the cases, which includes lung adenocarcinoma (LUAD) and lung squamous cell carcinoma (LUSC).^3^ Improved understanding of genomes and signaling pathways has revolutionized NSCLC therapy with improved outcomes in NSCLC patients,^4^, ^5^ but the survival rates for the disease remain low, especially for the advanced stages. Thus, there is an urgent need to further investigate the molecular mechanisms of lung cancer with a crucial goal of identifying reliable earlier diagnostic markers and new effective therapeutic targets.

Alternative splicing (AS) occurs for multiple different mRNA precursors, as it increases proteomic diversity from a limited gene repertoire.^6^ Up to 95% of the multiexon genes undergo AS in humans.^7^ AS has profound functional effects by changing the proteins encoded by mRNAs in various ways, including the production of protein variants with different biological functions.^8^ Dysregulation of AS affects essential biological processes and has been implicated in various ways in disease pathophysiology, raising the possibility of its use in novel therapeutics for diseases, including cancer.^9^, ^10^ In recent years, it has become clear that dysregulation of splicing could significantly contribute to cancer malignancies, and aberrant AS events have been found to be involved in various tumor biologies, including proliferation, invasion and metastasis, apoptosis, hypoxia, metabolism, angiogenesis and immune escape.^11^ Dysregulated AS has been considered another hallmark of cancer.^12^, ^13^ Splicing factors are pivotal regulators of AS, playing a crucial role in tumor AS dysregulation. Cancer cells often display aberrant AS profiles that are frequently caused by mutations or abnormal expressions of splicing factors.^14^ Thus, splicing factors might be novel potential therapeutic targets in cancer.

Plant homeodomain (PHD)–finger domain protein PHF5A, a critical splicing factor in AS regulation, is a subunit of the splicing factor 3b (SF3b) component of the U2 small nuclear ribonucleoprotein (U2 snRNP) splicing complex.^15^ PHF5A was found to act as a bridge protein to facilitate the interactions between the U2 snRNP complex and ATP–dependent helicases.^16^ During pre‐mRNA splicing, the complicated spliceosome recognized the branch point adenosine (BPA) by the binding pocket comprising PHF5A and SF3b subunit 1 (SF3B1), which could be targeted by splicing modulators, such as pladienolide, herboxidiene and spliceostatin.^17^ Strikoudis et al found that PHF5A could regulate the maintenance of pluripotency and cellular reprogramming in pluripotent embryonic stem cells (ESCs).^18^ Hubert et al^19^ certified that PHF5A was integral to the survival of glioblastoma multiforme (GBM) stem cells (GSCs) by mediating the recognition of specific exons with unusual C‐rich 3’ splice sites in thousands of essential genes. Zheng et al^20^ demonstrated that PHF5A acted as an oncogene to promote breast cancer progression by inhibiting apoptosis. However, knowledge of the splicing function of PHF5A is still limited, and the role of PHF5A in cancer is rarely investigated. Recently, Yang et al demonstrated that PHF5A functioned as a novel oncoprotein in LUAD.^21^ However, the biological functions of PHF5A in lung cancer still need to be confirmed, and its downstream molecular mechanisms need to be further explored.

In the present study, we explored the expression and role of PHF5A in NSCLC and confirmed its oncogenic biological functions in LUAD. We found that PHF5A promoted LUAD progression by regulating the AS of many essential genes, in particular several cell cycle–associated genes such as SKP2, CHEK2, ATR and apoptosis–associated genes such as API5 and BCL2L13.

## 2 MATERIALS AND METHODS

### 2.1 Patients and clinical specimens

For the patient cohort in the immunohistochemistry (IHC) study, paired tumor and adjacent normal tissues from a total of 126 patients with stage I‐III NSCLC were collected between January 1999 and September 2001. All patients had surgically proven primary NSCLC and underwent surgery before radiotherapy or chemotherapy at the National Cancer Center/National Clinical Research Center for Cancer/Cancer Hospital. Four tissue microarrays were constructed by incorporating one representative core of each tissue. The clinical and pathological characteristics of these patients are summarized in Supplementary Table S1. Samples were obtained with informed consent, and the study was approved by the medical ethics committee of the National Cancer Center/National Clinical Research Center for Cancer/Cancer Hospital.

### 2.2 IHC and scoring

IHC was performed according to the manufacturer's instructions using anti‐PHF5A (ab193115, Abcam, USA) antibody. Slides were evaluated independently by two pathologists. The staining intensity was graded as 0 (negative), 1 (low), 2 (moderate) or 3 (high), and the proportion of staining cells was evaluated as 0 (negative), 1 (<10%), 2 (10‐50%), 3 (51‐80%), or 4 (>80%). The intensity and proportion scores were multiplied to generate the IHC index. The expression level was considered low (IHC index <6) or high (IHC index ≥6).

### 2.3 Cell culture and transfection

H1299 and A549 cells were cultured in RPMI 1640 medium supplemented with 10% fetal bovine serum (FBS), 100 UI/ml penicillin and 100 UI/ml streptomycin (Gibco, USA). All cell lines were maintained in a humidified incubator at 37°C and 5% CO_2_.

Small interfering RNA (siRNA) and plasmid transfections were performed with Lipofectamine RNAiMAX Transfection Reagent (Invitrogen, USA) and Attractene Transfection Reagent (QIAGEN, USA), respectively. For silencing of PHF5A, three siRNA oligonucleotides (5′‐GGCUAAACAUCAUCCUGAUTT‐3′, 5′‐GCGCAUAUGUGAUGAGUGUTT‐3′ and 5′‐GGGUCUCUGAUGCCUAUUATT‐3′) and one negative control siRNA (5′‐UUCUCCGAACGUGUCACGUTT‐3′) were compounded by Sangon (Shanghai, China). For overexpression, the open reading frame (ORF) of PHF5A with C terminal Flag and His tag was compounded by Vigene (Shandong, China) and ligated into the pEnter vector; the empty vector was used as negative control.

### 2.4 RNA extraction and qRT‐PCR

Total RNA was isolated with the standard TRIzol‐based protocol (Invitrogen, USA). Quantitative real time PCR was performed on an ABI 7900HT RT‐PCR thermocycler (Life Technologies). Fold differences were calculated according to the 2^−∆∆Ct^ method and normalized against the endogenous expression of GAPDH. The gene specific primers were as follows: GAPDH (sense: 5′‐CCTGGTATGACAACGAATTTG‐3′, antisense: 5′‐CAGTGAGGGTCTCTCTCTTCC‐3′) and PHF5A (sense: 5′‐TGGTGTTGCCATCGGAAGAC‐3′, antisense: 5′‐CAGGGACGCACATAGGAGTC‐3′).

### 2.5 Western blot

Identical quantities of proteins were separated using sodium dodecyl sulfate–polyacrylamide gel electrophoresis and transferred onto nitrocellulose filter membranes. After incubation with antibodies specific for human GAPDH (CST) and PHF5A (ab116014, Abcam), the blots were incubated with horseradish peroxidase (HRP)–conjugated second antibody and were detected using an ImageQuant LAS 4000 (GE).

### 2.6 Cell proliferation and colony formation assays

For the cell proliferation assays, 1.5 × 10^3^ or 2.5 × 10^3^ cells per well were plated into 96‐well plates, and then the cell viabilities were tested every 24 hours for 72‐96 hours using the Cell Counting Kit 8 (CCK8; Dojindo). For colony formation assays, transfected cells (400 cells per well) were seeded into 6‐well plates and maintained in media containing 10% FBS. After 2 weeks, the colonies were fixed in methanol and stained with 0.1% crystal violet (Sigma), images of the colony formation assay results were scanned, and the clone number was determined using GeneSys software (Genecompany, China).

### 2.7 Transwell assays

Cells (5 × 10^4^ or 1 × 10^5^) in serum–free RPMI 1640 medium were plated into the upper chamber of 24‐well transwell inserts (Corning, 8.0 μm pores) that were either uncoated or coated with Matrigel for migration or invasion assay. The cells were then allowed to translocate toward medium containing 20% FBS for 24 hours. Cells on the lower side of the chamber were fixed, stained and counted in five different areas at 100‐fold magnification.

### 2.8 Cell cycle and cell apoptosis analysis

For cell cycle analysis, the harvested cells were fixed with 70% alcohol at 4°C overnight, digested in RNase at 37°C for 30 minutes, stained with propidium iodide (PI) for 20 minutes and analyzed with a BD flow cytometer (Becton Dickinson FACSCanto II). For cell apoptosis analysis, cells were harvested after a 24‐hour treatment with cisplatin, stained with FITC annexin V and PI in the dark, and assessed using a BD flow cytometer within 30 minutes.

### 2.9 RNA sequencing and AS analysis

Detailed library construction and RNA sequencing are provided in Supplementary Methods S1.

Differentially spliced genes were analyzed using rMATS, a new statistical method for the robust and flexible detection of differential AS from RNA–seq data.^22^ Percent spliced in (PSI) values quantifying alternative splicing event from zero to one were calculated.^23^ Differentially spliced genes between siRNA‐1 and siRNA‐NC groups with a false discovery rate (FDR) ≤0.05 were determined to be significantly differentially spliced genes.

For RT‐PCR validation, PCR primers (Supplementary Table S3) were designed (Primer Premier 5) to exons flanking predicted splicing events and were used to amplify the cDNA isoforms present before size separation on an agarose gel and detection using ethidium bromide.

### 2.10 Statistics

Statistical analyses were performed using Prism GraphPad version 6.0 (GraphPad Software Inc, San Diego, CA). The Chi square test was performed to determine the correlation between clinicopathological variables and PHF5A expression. Survival analysis was carried out using a log‐rank test. The significance of differences between groups was analyzed using two‐tailed Student's *t* test, and the results are expressed as the mean ± SD. Differences were considered significant when *P* < 0.05. *P < 0.05, **P < 0.01, ***P < 0.001 and ****P < 0.001.

## 3 RESULTS

### 3.1 An elevated level of PHF5A predicted poor prognosis in LUAD patients

**Figure 1:**
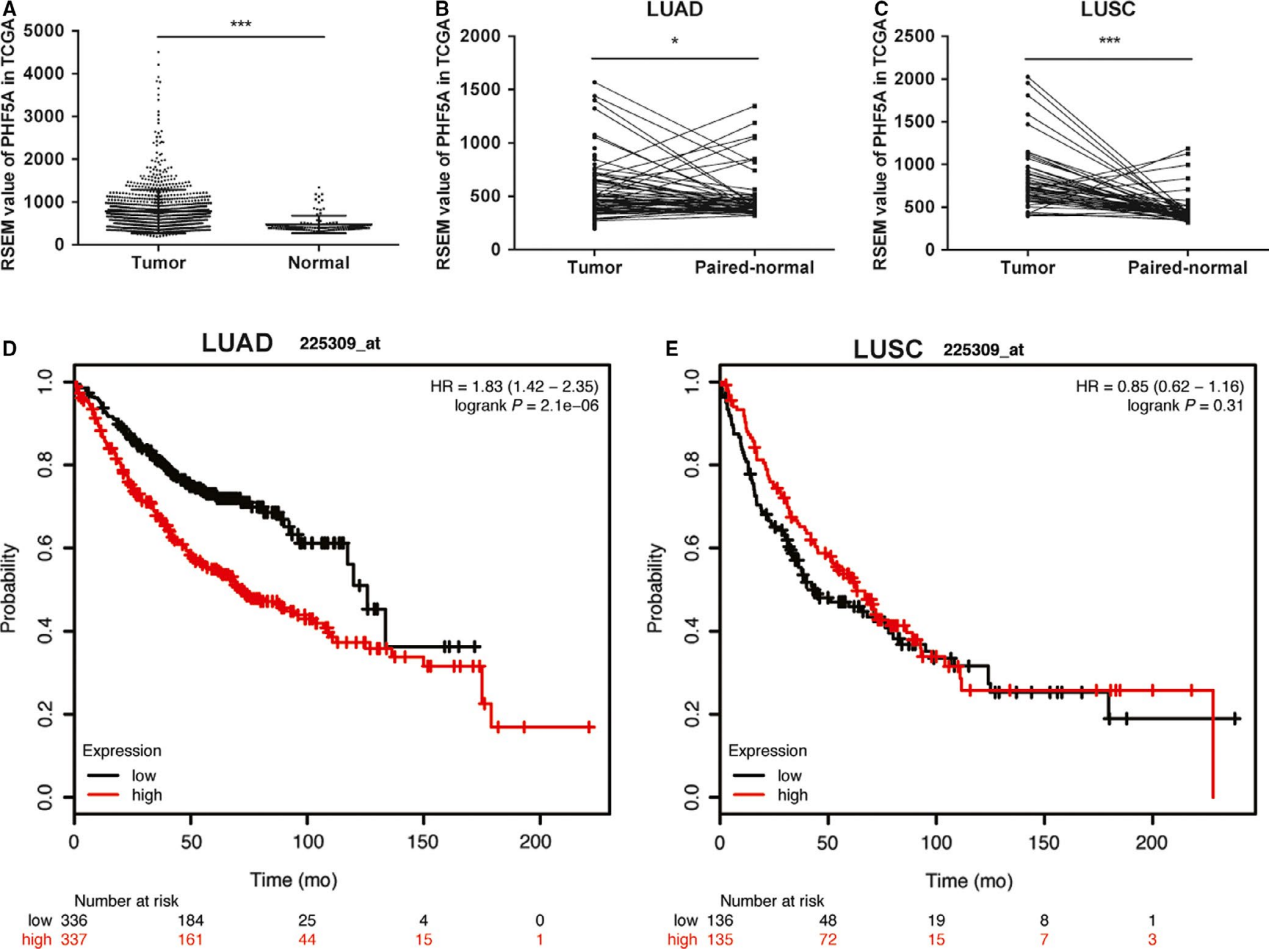
PHF5A upregulation is associated with poor patient survival in The Cancer Genome Atlas (TCGA) data sets. A, PHF5A expression was significantly increased in NSCLC tumor tissues (n = 1016) compared with normal tissues (n = 110). B and C, PHF5A expression was significantly increased in lung adenocarcinoma (LUAD) (B, n = 58) and lung squamous cell carcinoma (LUSC) (C, n = 51) tumor tissues compared with paired adjacent normal tissues. D and E, A high mRNA level of PHF5A was significantly associated with shorter OS in LUAD patients (D) but not LUSC patients (E). *P < 0.05; ***P < 0.001

Analysis of NSCLC cohorts from The Cancer Genome Atlas (TCGA) data sets revealed that PHF5A expression was significantly higher in NSCLC tumor tissues (n = 1016) than in normal tissues (n = 110) (Figure 1A), and compared with the paired adjacent normal tissues, tumor tissues also showed upregulated expression of PHF5A in both the LUAD (Figure 1B) and LUSC (Figure 1C) cohorts. Recently, when using gene expression levels calculated from TCGA LUAD and LUSC level 3 RNA–seq data, PHF5A was found to be significantly negatively–associated with patient OS in LUAD.^24^ Therefore, we verified the associations between PHF5A expression and NSCLC patient survival using data sets provided by the Kaplan‐Meier plotter.^25^ Higher PHF5A mRNA expression was significantly correlated with shorter OS in LUAD patients (n = 673) (Figure 1D) but not in LUSC patients (n = 271) (Figure 1E).

**Figure 2:**
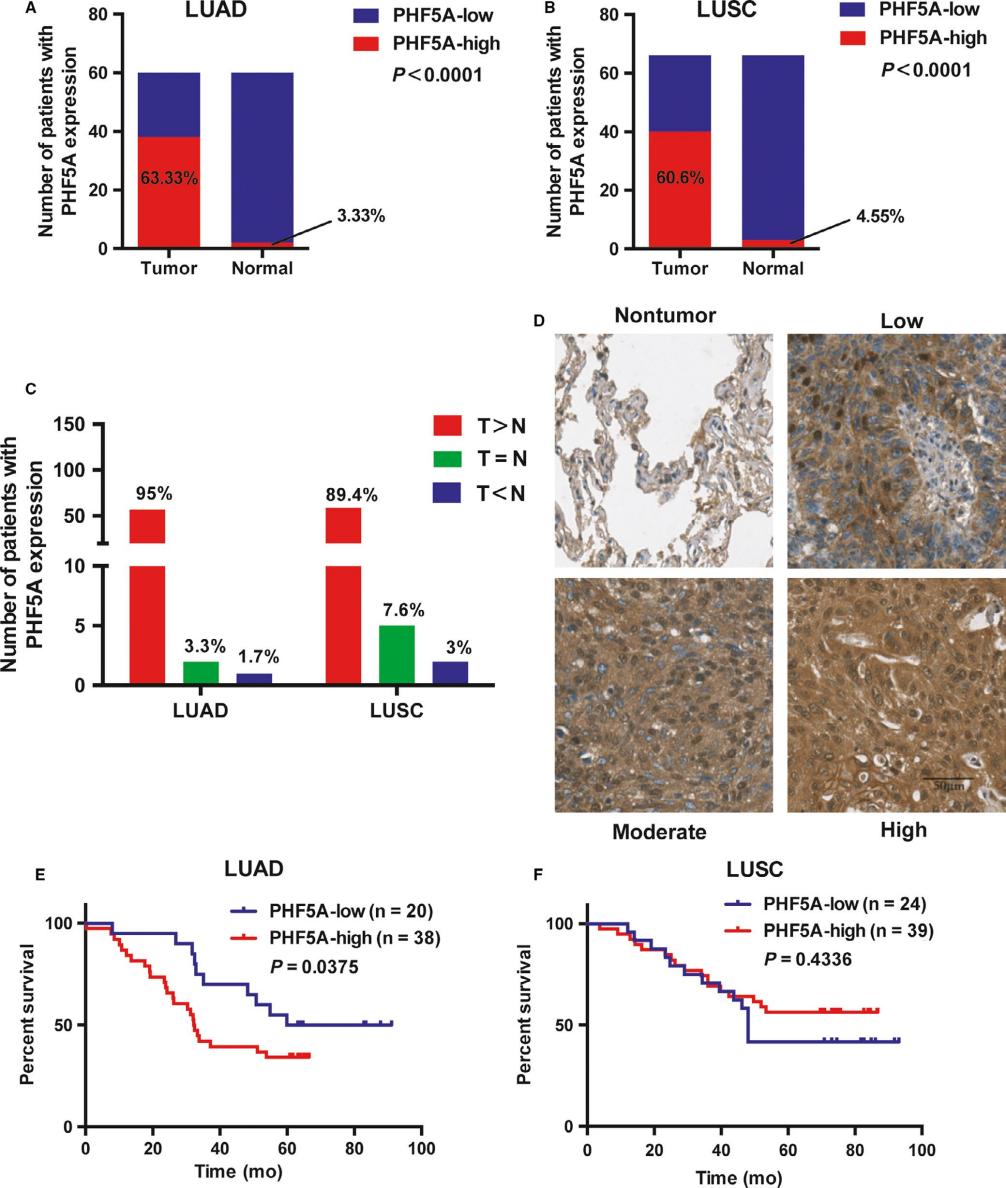
The expression of PHF5A is significantly upregulated in both lung adenocarcinoma (LUAD) and lung squamous cell carcinoma (LUSC) patient samples. A and B, The percentage of PHF5A‐low and PHF5A‐high staining in tumor and paired normal tissues in LUAD (A, n = 60) and LUSC (B, n = 66) patients. C, The number and percentage of patients with higher, equal or lower PHF5A staining in LUAD and LUSC tumor tissues compared with paired normal tissues. T: tumor tissue; N: paired normal tissue. D, Representative IHC images of PHF5A staining in NSCLC tumor tissues and paired normal tissues. E and F, Kaplan‐Meier plots based on the expression level of PHF5A measured by IHC in 58 LUAD (E) and 63 LUSC (F) patients

To verify the relationship between the expression level of PHF5A and the clinical outcomes of NSCLC patients, IHC was conducted in four tissue microarrays containing 126 NSCLC tissue specimens and their matched adjacent normal tissues. In LUAD, the expression level of PHF5A was stained highly in approximately 63.33% (38/60) of the tumor tissues but in only 3.33% (2/60) of the nontumor tissues (Figure 2A); in LUSC, the percentage of high expression of PHF5A was 60.6% (40/66) and 4.55% (3/66) in tumor and normal tissues, respectively (Figure 2B). When comparing the staining results of tumor tissues with those of their paired nontumor tissues, approximately 95% (57/60) and 89.4% (59/66) of the tumor tissues exhibited increased PHF5A expression (T > N) in LUAD and LUSC, respectively (Figure 2C). Clinicopathological analysis showed a significant correlation between the expression level of PHF5A and TNM stage in patients with LUAD but not LUSC (Supplementary Table S1). Moreover, according to Kaplan‐Meier analysis, patients with high expression levels of PHF5A had a shorter OS than those with low PHF5A expression in LUAD (Figure 2E) but not in LUSC (Figure 2F), which was concordant with the results obtained using TCGA database.

### 3.2 Knockdown of PHF5A inhibited LUAD cell proliferation, invasion and migration in vitro

**Figure 3:**
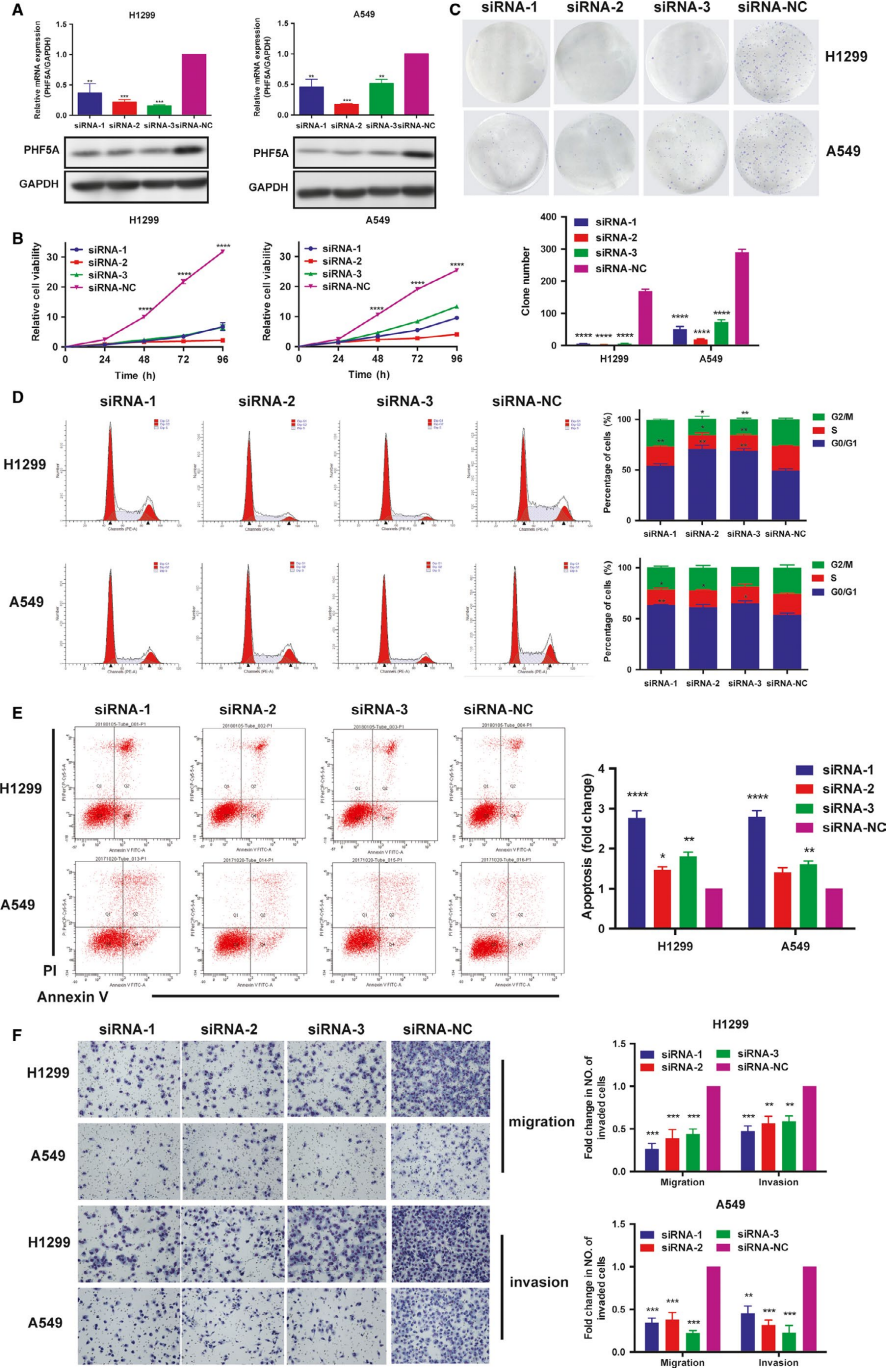
Downregulation of PHF5A inhibited lung adenocarcinoma (LUAD) cell proliferation, invasion and migration. A, The knockdown efficiency of PHF5A was examined by qRT‐PCR and western blot. GAPDH was used as a control. B, The proliferation ability of H1299 and A549 cell lines transfected with siRNAs was measured by CCK8 assay. C, Clone formation assays in PHF5A knockdown H1299 and A549 cell lines and control cells. D, Cell cycle analysis in PHF5A knockdown H1299 and A549 cell lines and control cells. E, Cisplatin–induced apoptosis in PHF5A knockdown H1299 and A549 cell lines and control cells. F, The migration and invasion ability in PHF5A knockdown H1299 and A549 cell lines and control cells. Data are expressed as the means ± SD. *P < 0.05, **P < 0.01, ***P < 0.001, ****P < 0.0001. Student's *t* test

To investigate the biological function of PHF5A in LUAD, we constructed three siRNAs targeting PHF5A (siRNA‐1, siRNA‐2, and siRNA‐3) and one control siRNA (siRNA‐NC). The RNA and protein levels of PHF5A were both markedly decreased after transfection with specific PHF5A siRNAs in H1299 and A549 LUAD cell lines compared with control siRNA‐NC (Figure 3A). Downregulation of PHF5A caused a significant decrease in cell proliferation (Figure 3B) and colony formation in H1299 and A549 (Figure 3C). Fluorescence–activated cell sorting (FACS) analysis showed that, compared with the control condition, downregulation of PHF5A expression level resulted in an increased number of G0/G1 cells (Figure 3D) and enhanced apoptosis of H1299 and A549 cells after 24‐hour treatment with cisplatin (Figure 3E). In addition, decreased expression of PHF5A significantly inhibited the invasion and migration ability of H1299 and A549 cells (Figure 3F). Thus, PHF5A positively regulated proliferation, invasion and migration of LUAD cells, acting as an oncogenic gene in LUAD.

### 3.3 Overexpression of PHF5A promoted LUAD cell proliferation, invasion and migration in vitro

**Figure 4:**
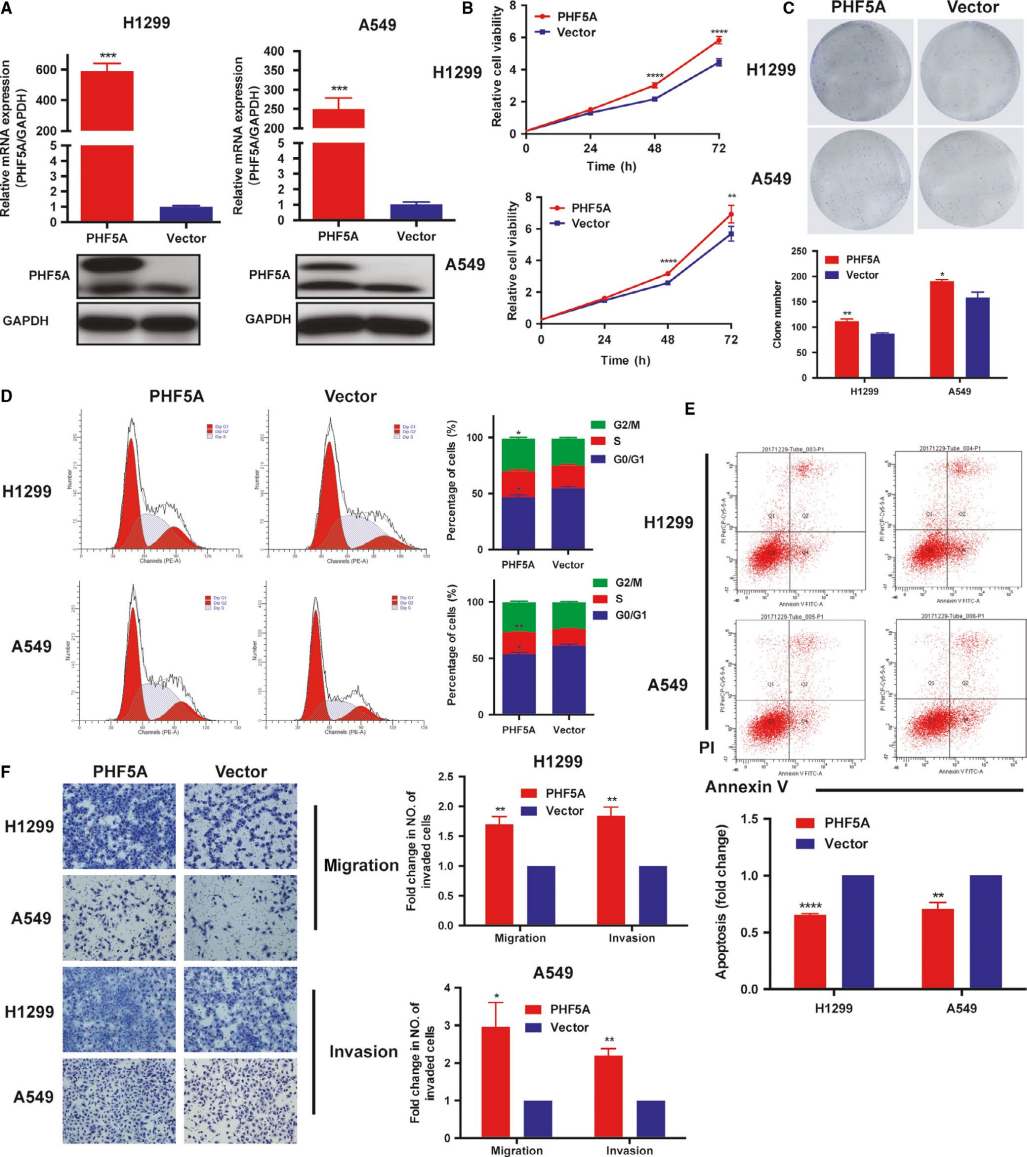
Overexpression of PHF5A promoted lung adenocarcinoma (LUAD) cell proliferation, invasion and migration. A, The overexpression efficiency of PHF5A was examined by qRT‐PCR and western blot. GAPDH was used as the control. B, The proliferation ability of H1299 and A549 cell lines transfected with PHF5A and control vectors was measured by CCK8 assay. C, Clone formation assays in PHF5A overexpressed H1299 and A549 cell lines and control cells. D, Cell cycle analysis in PHF5A–overexpressed H1299 and A549 cell lines and control cells. E, Cisplatin–induced apoptosis in PHF5A–overexpressed H1299 and A549 cell lines and control cells. F, The migration and invasion ability in PHF5A–overexpressed H1299 and A549 cell lines and control cells. Data are expressed as the means ± SD. *P < 0.05, **P < 0.01, ***P < 0.001, ****P < 0.0001. Student's *t* test

To verify the oncogenic role of PHF5A in LUAD, we established cell models with PHF5A overexpression using H1299 and A549 cell lines. The expression level of PHF5A was greatly increased after transfection with a PHF5A overexpression vector (Figure 4A). As expected, LUAD cells overexpressing PHF5A exhibited a significantly higher proliferation rate and colony formation capacity than those transfected with the empty vector (Figure 4B,C). FACS analysis showed that overexpression of PHF5A promoted the cell cycle transition from G0/G1 to S phase (Figure 4D) and inhibited cisplatin–induced cell apoptosis (Figure 4E). Furthermore, elevated expression of PHF5A significantly promoted the invasion and migration ability of H1299 and A549 cells (Figure 4F). These results supported the findings obtained during PHF5A knockdown.

### 3.4 Knockdown of PHF5A induced genome‐wide alternative splicing events

**Figure 5:**
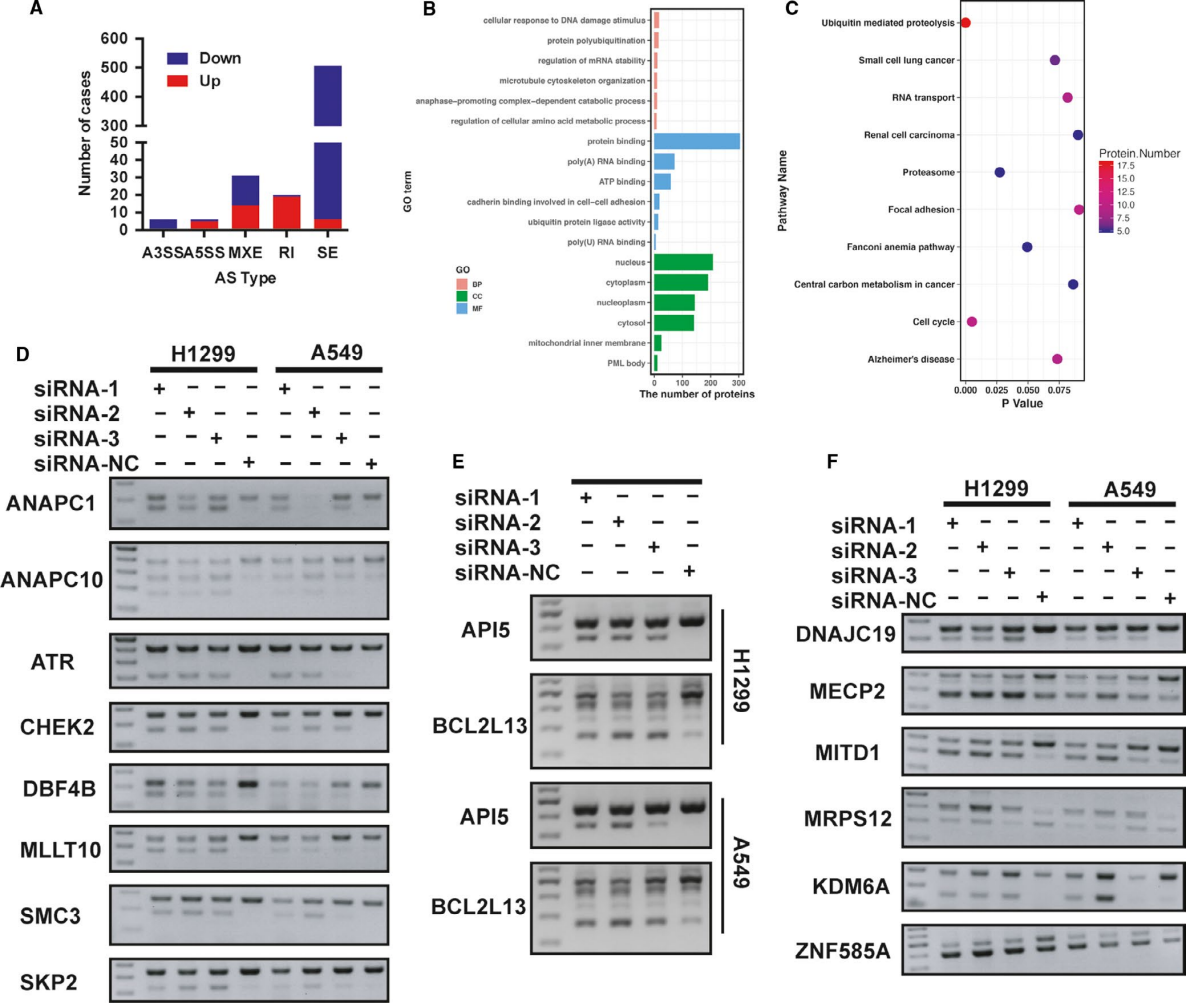
Knockdown of PHF5A–induced genome–wide alternative splicing (AS) events. A, The number of significantly differentially spliced events in five AS types. B, GO enrichment analysis in differentially spliced genes. C, KEGG pathway enrichment analysis in differentially spliced genes. D, Validation of AS events in cell cycle–related genes using RT‐PCR. E, Validation of AS events in apoptosis–related genes using RT‐PCR. F, Validation of AS events in other differentially spliced genes using RT‐PCR

Because PHF5A is a core subunit of the U2 snRNP splicing complex, it acts as a splicing factor in the regulation of gene AS. We investigated whether it promoted lung cancer progression via regulation of the AS of some essential genes. Therefore, we conducted mRNA sequencing in H1299 and A549 cell lines transfected with PHF5A targeting siRNA‐1 and control siRNA‐NC. Overall, we identified that a total of 569 differentially spliced events existed in both H1299 and A549 cell lines, with 44 AS events whose PSI was upregulated and 525 AS events whose PSI was downregulated (Supplementary Table S2). Among all of these differentially spliced events, various types of AS events could be regulated by PHF5A, including 506 skipped exons (SE), which was the most common splice type, 31 mutually exclusive exons (MXE), 20 retained introns (RI), 6 alternative 5’ exons (A5SSs) and 6 alternative 3’ exons (A3SSs) (Figure 5A, Supplementary Table S2).

To further investigate the biological functions and molecular mechanisms of these differentially spliced genes regulated by PHF5A, we conducted Gene Ontology (GO) and Kyoto Encyclopedia of Genes and Genomes (KEGG) pathway analysis. Interestingly, cellular response to DNA damage stimulus and cell cycle pathways were pivotal signaling pathways affected by these differentially spliced genes in GO and KEGG enrichment analysis, respectively (Figure 5B,C), which indicated the potential role of PHF5A in cell cycle and apoptosis pathways. Because the in vitro experiments also demonstrated that knockdown of PHF5A inhibited LUAD cell proliferation partially by inducing G0/G1 cell cycle arrest and promoting cell apoptosis, we focused on cell cycle and apoptosis pathways to elucidate the underlying molecular mechanisms of PHF5A. We utilized RT‐PCR and agarose gel electrophoresis to validate the most common differentially spliced AS events as well as the cell cycle–associated and apoptosis–associated spliced genes in H1299 and A549 cell lines transfected with PHF5A targeting siRNA‐1, siRNA‐2, siRNA‐3 and control siRNA‐NC. Notably, we validated that PHF5A could regulate the AS of many cell cycle–related genes including ANAPC1, ANAPC10, ATR, CHEK2, DBF4B, MLLT10, SMC3 and SKP2 (Figure 5D), apoptosis–associated genes including API5 and BCL2L13 (Figure 5E) as well as some other pathway genes including DNAJC19, MECP2, MITD1, MRPS12, KDM6A, and ZNF585A (Figure 5F). We concluded that PHF5A function as a global splicing factor affected the biological behavior of lung cancer cells by regulating the AS of many downstream genes, especially cell cycle–associated and apoptosis–associated genes.

### 3.5 Small molecular inhibitor of PHF5A inhibited cell proliferation and induced alternative splicing of essential genes

**Figure 6:**
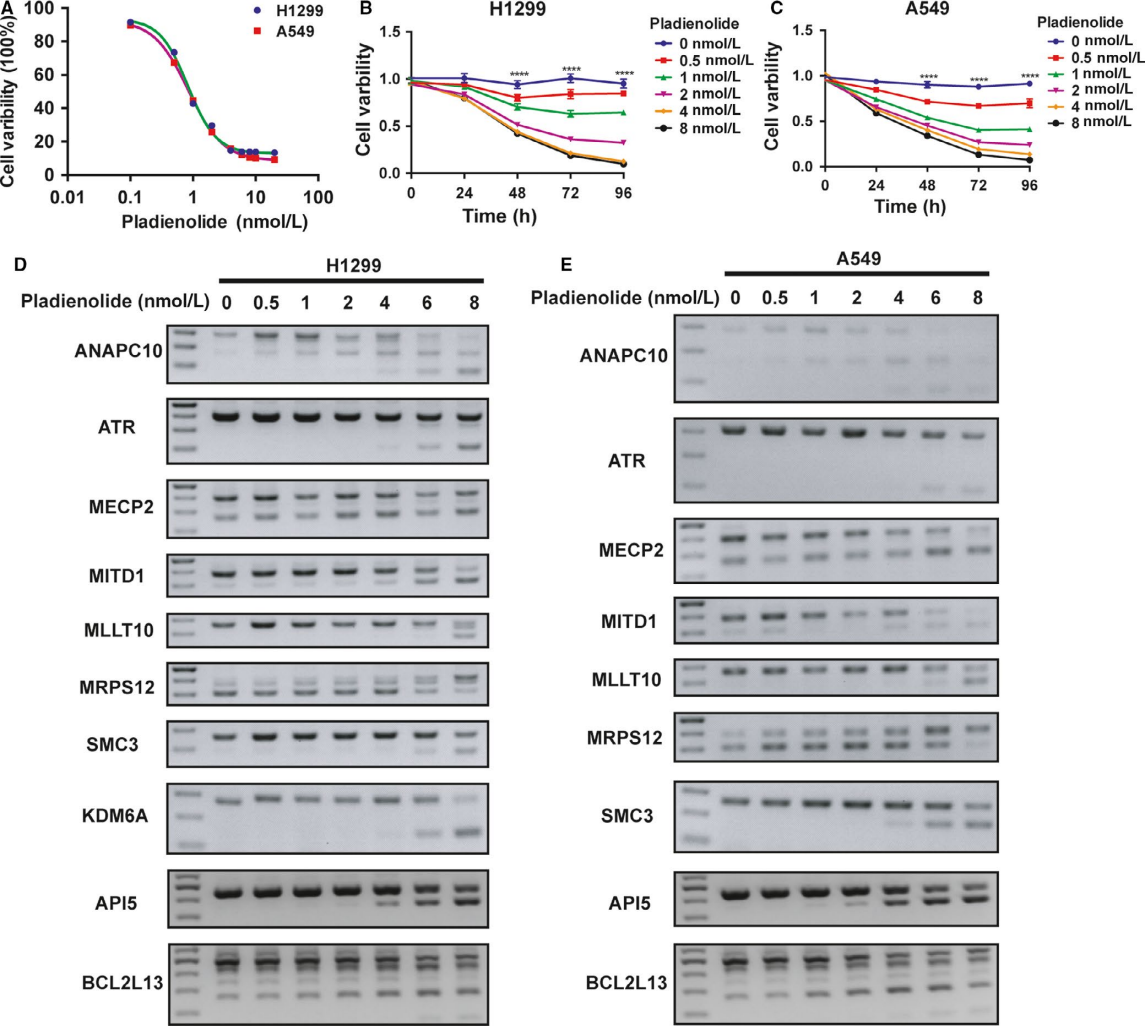
Inhibitor of PHF5A inhibited lung adenocarcinoma (LUAD) cell proliferation and induced similar alternative splicing (AS) events. A, The IC50 curve of pladienolide in H1299 and A549 cell lines. B and C, CCK8 assays in H1299 (B) and A549 (C) cell lines treated with different concentrations of pladienolide. D and E, Validation of AS events in partial genes in H1299 (D) and A549 (E) cell lines treated with different concentrations of pladienolide. ****P < 0.0001

Since PHF5A played an important role in lung cancer progression, we speculated whether PHF5A could act as a new drug target or whether an inhibitor of PHF5A could exert an antitumor effect. Pladienolide, a small molecular compound identified as a splicing modulator that targets SF3B1 in the SF3b subcomplex, has been recently discovered to act at the BPA binding pocket defined by the PHF5A–SF3b complex.^17^ Therefore, we used pladienolide to treat H1299 and A549 cell lines. We found that pladienolide inhibited cell proliferation in a dose–dependent manner (Figure 6A‐C). More importantly, we found that pladienolide induced AS events similarly to PHF5A knockdown in a dose–dependent manner, including splicing of ANAPC10, ATR, MECP2, MITD1, MLLT10, MRPS12, SMC3, KDM6A, API5 and BCL2L13 (Figure 6D‐E). These findings indicated that PHF5A indeed played a pivotal role in AS and could be a potential antitumor drug target in the future.

## 4 DISCUSSION

In the present study, we demonstrated that PHF5A functioned as an oncoprotein in the progression of LUAD by regulating AS of various genes throughout the genome. The mRNA and protein levels of PHF5A were significantly upregulated in both TCGA mRNA–seq data set and our own NSCLC patient tumor samples, and the expression level of PHF5A was significantly negatively correlated with patient outcomes in LUAD but not in LUSC. Knockdown of PHF5A in LUAD cell lines inhibited cell proliferation, migration and invasion, induced G0/G1 cell cycle arrest and promoted cisplatin–induced apoptosis. Overexpression of PHF5A showed the opposite results, validating the oncogenic role of PHF5A in LUAD. Further exploration of the underlying molecular mechanisms found that PHF5A played a vital role in the AS of many essential genes, including cell cycle–related and apoptosis–associated genes. Moreover, pladienolide, a small molecular inhibitor of PHF5A, inhibited lung cancer cell proliferation in a dose–dependent manner and induced similar downstream gene changes. All of these data supported our conclusion that PHF5A has pleiotropic effects on LUAD cell proliferation, migration and invasion and thus has oncogenic activity in LUAD. Therefore, PHF5A might be a potential antitumor drug target in the future.

AS, an important posttranscriptional process that produces different mature mRNAs from a single gene, has been widely validated to be involved in the biology of various tumors.^11^ Splicing factor, a core regulator in AS, has been found to be mutated or dysregulated in many tumors, affecting the AS of pre‐mRNAs globally.^14^ PHF5A is a key splicing factor in SF3b, which is a subunit of the U2 snRNP splicing complex.^26^ The biological function and the molecular mechanisms of PHF5A in tumors have been rarely investigated. In GBM, PHF5A was discovered to be integral for the survival of GSCs by mediating the recognition of specific exons with unusual C‐rich 3’ splice sites in thousands of essential genes.^19^ Recently, using in vivo CRISPR screening, Zheng et al identified PHF5A as a key splicing factor involved in breast cancer progression; they found that PHF5A was required for SF3b spliceosome stability and linked the complex to histones, and the PHF5A–SF3b complex modulated AS changes in apoptotic signaling pathways.^20^ However, there was no research investigating the role of PHF5A in lung cancer until a few months ago. Yang et al recently demonstrated that PHF5A was also an oncoprotein in LUAD.^21^ However, the biological function of PHF5A in lung cancer and the exact molecular pathways still need to be further explored.

In our study, we first analyzed the mRNA expression of PHF5A using TCGA NSCLC level 3 RNA–seq data. We found that the mRNA expression level of PHF5A was significantly increased in both LUAD and LUSC tumor tissues compared with normal tissues. In addition, high expression of PHF5A mRNA was significantly correlated with shorter OS in LUAD patients but not in LUSC patients, as shown in the Kaplan‐Meier plotter. Furthermore, using IHC in our own NSCLC tissue microarrays, we validated that the protein level of PHF5A was also significantly upregulated in both LUAD and LUSC tumor tissues compared with paired nontumor tissues, and the high expression of PHF5A protein was significantly correlated with poor survival in LUAD patients but not LUSC patients, which was consistent with the findings obtained using TCGA data sets. The difference in the correlations between the expression level of PHF5A and patient outcomes in LUAD and LUSC might be due to the different molecular characterizations in different pathological subtypes. An in‐depth understanding of the reasons underlying this difference remains to be determined.

Because of the clear negative correlation between the expression of PHF5A and LUAD patient OS, we chose two LUAD cell lines, H1299 and A549, to carry out further research. Knockdown of PHF5A in H1299 and A549 significantly inhibited cell proliferation, clone formation ability, migration and invasion ability; promoted G0/G1 cell cycle arrest; and induced cisplatin–induced apoptosis in vitro. In contrast, overexpression of PHF5A promoted cell proliferation, clone formation ability, migration and invasion ability, prompted cells to go through the G0/G1 phase of the cell cycle and inhibited cisplatin–induced apoptosis.

To further evaluate the underlying molecular mechanisms of PHF5A, we conducted next–generation sequencing using RNA extracted from H1299 and A549 cell lines transfected with PHF5A targeting siRNA‐1 and control siRNA‐NC. Interestingly, we found up to 569 differentially spliced AS events in both H1299 and A549 cell lines. Bioinformatic analysis implied that most differentially spliced genes were enriched in the cellular response to DNA damage stimulus process and the cell cycle pathways. We used RT‐PCR and agarose gel electrophoresis to validate the results of RNA‐seq, and some important genes were verified, including ANAPC1, ANAPC10, ATR, CHEK2, DBF4B, MLLT10, SMC3, SKP2, DNAJC19, MECP2, MITD1, MRPS12, KDM6A, ZNF585A, API5 and BCL2L13. This result indicated that PHF5A could affect the AS of many genes globally in LUAD just like its effect in GBM^19^ and breast cancer.^20^ Most of these validated genes were found to be closely related to tumor biology and played important roles in tumor development and progression. For example, SKP2 is a subunit of the Skp1/Cullin/F‐box protein (SCF) complex, which is a ubiquitin ligase mainly targeting the CDK inhibitor p27 during S phase, leading to the degradation of p27 to promote G0/G1 cell cycle progression.^27^ Alternatively, SKP2 suppresses p53‐dependent apoptosis by outcompeting p53 for binding to p300, thereby perturbing p300–mediated p53 acetylation and stabilization.^28^ More importantly, SKP2 was found to be frequently increased in many human cancers and played an oncogenic role in tumorigenesis^29^; inhibition of SKP2 has emerged as a promising anticancer strategy.^30^ CHEK2 is a key protein in the regulation of the G2/M cell cycle checkpoint^31^ and was discovered to be a multiorgan cancer susceptibility gene, contributing to the development of various cancers, including breast, colorectal, prostate, ovarian, thyroid and kidney cancer.^32^, ^33^ ATR is an essential kinase that is activated in S phase to orchestrate the multifaceted response to DNA replication stress, ensuring the completion of DNA replication and maintaining the integrity of the genome.^34^ Combining genotoxic chemotherapies with ATR inhibitors in antitumor therapy could induce high loads of replication stress in cancer cells and force cells with unreplicated genomes into mitosis, leading to mitotic catastrophe and p53–independent cell death.^35^ Therefore, several ATR inhibitors are entering clinical trials, presenting a new strategy for cancer therapy.^35^ Collectively, we could conclude that PHF5A promoted LUAD progression by regulating the AS of many essential genes.

In addition, when using PHF5A small molecular inhibitor pladienolide to treat H1299 and A549 cell lines, we found that pladienolide significantly inhibited LUAD cell proliferation in a dose–dependent manner and induced AS events similar to those obtained in PHF5A knockdown, indicating that PHF5A might be a potential drug target in lung cancer treatment.

Compared with Yang's work,^21^ our work had several different results and conclusions. First, we analyzed the expression of PHF5A in LUSC patients using both TCGA data sets and our own tumor tissue samples and found that PHF5A was upregulated in LUSC patients but was not significantly correlated with patient outcomes, and this finding differed from those observed in LUAD. Second, Yang et al just studied the biological effect of PHF5A knockdown in H1299 and H1975, whereas we additionally completed functional experiments of PHF5A overexpression and further confirmed the oncogenic role of PHF5A in LUAD. Third, when we investigated the underlying molecular mechanisms, we focused on AS, which is the main biological function of PHF5A, rather than the downstream protein level changes. Finally, we used an inhibitor of PHF5A to further validate the AS function of PHF5A, highlighting its potential value in clinical application.

Taken together, our findings demonstrated that PHF5A acts as a key regulator in the AS of many essential genes, thus promoting the development and progression of LUAD. The findings of this study provide a novel perspective in understanding the pathogenesis of lung cancer and might have significant implications with respect to the relationship between AS and lung cancer tumorigenesis. PHF5A might serve as a future potential drug target with promising anticancer therapeutic effects.

## CONFLICT OF INTEREST

The authors declare no potential conflicts of interest.

## References

[1] Chen W , Zheng R , Baade PD , et al. Cancer statistics in China, 2015. CA Cancer J Clin. 2016;66(2):115‐132. 26808342 10.3322/caac.21338

[2] Torre LA , Bray F , Siegel RL , Ferlay J , Lortet‐Tieulent J , Jemal A . Global cancer statistics, 2012. CA Cancer J Clin. 2015;65(2):87‐108. 25651787 10.3322/caac.21262

[3] Travis WD , Brambilla E , Nicholson AG , et al. The 2015 World Health Organization classification of lung tumors: impact of genetic, clinical and radiologic advances since the 2004 classification. J Thorac Oncol. 2015;10(9):1243‐1260. 26291008 10.1097/JTO.0000000000000630

[4] Julia Rotow T . Understanding and targeting resistance mechanisms in NSCLC. Nat Rev Cancer. 2017;17:637‐658. 29068003 10.1038/nrc.2017.84

[5] Chen Z , Fillmore CM , Hammerman PS , Kim CF , Wong KK . Non‐small‐cell lung cancers: a heterogeneous set of diseases. Nat Rev Cancer. 2014;14(8):535‐546. 25056707 10.1038/nrc3775 PMC5712844

[6] Nilsen TW , Graveley BR . Expansion of the eukaryotic proteome by alternative splicing. Nature. 2010;463(7280):457‐463. 20110989 10.1038/nature08909 PMC3443858

[7] Pan Q , Shai O , Lee LJ , Frey BJ , Blencowe BJ . Deep surveying of alternative splicing complexity in the human transcriptome by high‐throughput sequencing. Nat Genet. 2008;40(12):1413‐1415. 18978789 10.1038/ng.259

[8] Kelemen O , Convertini P , Zhang Z , et al. Function of alternative splicing. Gene. 2013;514(1):1‐30. 22909801 10.1016/j.gene.2012.07.083 PMC5632952

[9] Gamazon ER , Stranger BE . Genomics of alternative splicing: evolution, development and pathophysiology. Hum Genet. 2014;133(6):679‐687. 24378600 10.1007/s00439-013-1411-3

[10] Padgett RA . New connections between splicing and human disease. Trends Genet. 2012;28(4):147‐154. 22397991 10.1016/j.tig.2012.01.001 PMC3319163

[11] David CJ , Manley JL . Alternative pre‐mRNA splicing regulation in cancer: pathways and programs unhinged. Genes Dev. 2010;24(21):2343‐2364. 21041405 10.1101/gad.1973010 PMC2964746

[12] Oltean S , Bates DO . Hallmarks of alternative splicing in cancer. Oncogene. 2014;33(46):5311‐5318. 24336324 10.1038/onc.2013.533

[13] Aberrant ML . Alternative splicing is another hallmark of cancer. Int J Cell Biol. 2013;2013:463786. 24101931 10.1155/2013/463786 PMC3786539

[14] Srebrow A , Kornblihtt AR . The connection between splicing and cancer. J Cell Sci. 2006;119(Pt 13):2635‐2641. 16787944 10.1242/jcs.03053

[15] Trappe R , Ahmed M , Gläser B , et al. Identification and characterization of a novel murine multigene family containing a PHD‐finger‐like motif. Biochem Biophys Res Commun. 2002;293(2):816‐826. 12054543 10.1016/S0006-291X(02)00277-2

[16] Rzymski T , Grzmil P , Meinhardt A , Wolf S , Burfeind P . PHF5A represents a bridge protein between splicing proteins and ATP‐dependent helicases and is differentially expressed during mouse spermatogenesis. Cytogenet Genome Res. 2008;121(3–4):232‐244. 18758164 10.1159/000138890

[17] Teng T , Tsai J , Puyang X , et al. Splicing modulators act at the branch point adenosine binding pocket defined by the PHF5A‐SF3b complex. Nat Commun. 2017;8:15522. 28541300 10.1038/ncomms15522 PMC5458519

[18] Strikoudis A , Lazaris C , Trimarchi T , et al. Regulation of transcriptional elongation in pluripotency and cell differentiation by the PHD‐finger protein Phf5a. Nat Cell Biol. 2016;18(11):1127‐1138. 27749823 10.1038/ncb3424 PMC5083132

[19] Hubert Cg , Bradley Rk , Ding Y , et al. Genome‐wide RNAi screens in human brain tumor isolates reveal a novel viability requirement for PHF5A. Genes Dev. 2013;27(9):1032‐1045. 23651857 10.1101/gad.212548.112 PMC3656321

[20] Zheng Y‐Z , Xue M‐Z , Shen H‐J , et al. phf5a epigenetically inhibits apoptosis to promote breast cancer progression. Cancer Res. 2018;78(12):3190‐3206. 29700004 10.1158/0008-5472.CAN-17-3514

[21] Yang Y , Zhu J , Zhang T , et al. PHD‐finger domain protein 5A functions as a novel oncoprotein in lung adenocarcinoma. J Exp Clin Cancer Res. 2018;37(1):65. 29566713 10.1186/s13046-018-0736-0 PMC5863814

[22] Shen S , Park JW , Z‐x Lu , et al. Robust and flexible detection of differential alternative splicing from replicate RNA‐Seq data. Proc Natl Acad Sci U S A. 2014;111(51):E5593‐E5601. 25480548 10.1073/pnas.1419161111 PMC4280593

[23] Ryan MC , Cleland J , Kim R , Wong WC , Weinstein JN . SpliceSeq: a resource for analysis and visualization of RNA‐Seq data on alternative splicing and its functional impacts. Bioinformatics. 2012;28(18):2385‐2387. 22820202 10.1093/bioinformatics/bts452 PMC3436850

[24] Li Y , Sun N , Lu Z , et al. Prognostic alternative mRNA splicing signature in non‐small cell lung cancer. Cancer Lett. 2017;393:40‐51. 28223168 10.1016/j.canlet.2017.02.016

[25] Gyorffy B , Budczies J , Lanczky A . Online survival analysis software to assess the prognostic value of biomarkers using transcriptomic data in non‐small‐cell lung cancer. PLoS One. 2013;8(12):e82241. 24367507 10.1371/journal.pone.0082241 PMC3867325

[26] Will CL , Urlaub H , Achsel T , Gentzel M , Wilm M , Lührmann R . Characterization of novel SF3b and 17S U2 snRNP proteins, including a human Prp5p homologue and an SF3b DEAD‐box protein. EMBO J. 2002;21(18):4978‐4988. 12234937 10.1093/emboj/cdf480 PMC126279

[27] Hiroyuk Imaki KN , Delehouzee S , Handa H , Kitagawa M , Kamura T , Nakayama KI . Cell Cycle‐dependent Regulation of the Skp2 Promoter by GA‐binding protein. Cancer Res. 2003;63:4607‐4613. 12907639

[28] Kitagawa M , Lee SH , McCormick F . Skp2 suppresses p53‐dependent apoptosis by inhibiting p300. Mol Cell. 2008;29(2):217‐231. 18243116 10.1016/j.molcel.2007.11.036

[29] Hershko DD . Oncogenic properties and prognostic implications of the ubiquitin ligase Skp2 in cancer. Cancer. 2008;112(7):1415‐1424. 18260093 10.1002/cncr.23317

[30] Lee Y , Lim HS . Skp2 inhibitors: novel anticancer strategies. Curr Med Chem. 2016;23:1‐18. 10.2174/0929867323666160510122624 27160538

[31] Chaturvedi P , Eng WK , Zhu Y , et al. Mammalian Chk2 is a downstream e€ector of the ATM‐dependent DNA damage checkpoint pathway. Oncogene. 1999;18:4047‐4054. 10435585 10.1038/sj.onc.1202925

[32] Liu C , Wang Q‐S , Wang Y‐J . The CHEK2 I157T variant and colorectal cancer susceptibility: a systematic review and meta‐analysis. Asian Pac J Cancer Prev. 2012;13(5):2051‐2055. 22901170 10.7314/apjcp.2012.13.5.2051

[33] Zannini L , Delia D , Buscemi G . CHK2 kinase in the DNA damage response and beyond. J Mol Cell Biol. 2014;6:442‐457. 25404613 10.1093/jmcb/mju045 PMC4296918

[34] Saldivar JC , Cortez D , Cimprich KA . The essential kinase ATR: ensuring faithful duplication of a challenging genome. Nat Rev Mol Cell Biol. 2017;18(10):622‐636. 28811666 10.1038/nrm.2017.67 PMC5796526

[35] Lecona E , Fernandez‐Capetillo O . Targeting ATR in cancer. Nat Rev Cancer. 2018;18(9):586‐595. 29899559 10.1038/s41568-018-0034-3

