# Supplementary material for: PHD finger protein 5A promoted lung adenocarcinoma progression via alternative splicing
Source: Cancer Med. 2019 Apr 1;8(5):2429–41. doi: 10.1002/cam4.2115 (PMC6536992; doi:10.1002/cam4.2115)
Supplement: Supplementary file 2 [file CAM4-8-2429-s002.docx]

**Supplementary methods: Library construction and RNA sequencing**

Total RNA from H1299 and A549 cells after transfection with PHF5A-targeting siRNA-1 or siRNA-NC was isolated to perform RNA sequencing. Nanodrop, Qubit 2.0, Agilent 2100, and electrophoresis methods were used to detect the purity, concentration, integrity, and genomic DNA contamination, respectively, of RNA samples to ensure long noncoding RNA sequencing were of high quality. The epicenter Ribo-ZeroTM kit was used to remove sample rRNA. Subsequently, fragmentation, first cDNA synthesis, and second cDNA synthesis were carried out, and the cDNA was purified using AMPure XP beads. After fragmentation size selection, the U chain was degraded, and finally, the cDNA library was obtained by PCR enrichment. After the library quality assessment, the library was sequenced on the Illumina HiSeq platform.

**Supplementary Table S1. The relationship between PHF5A expression and clinicopathological parameters in LUAD and LUSC patients.**

|  | LUAD | | | LUSC | | |
| --- | --- | --- | --- | --- | --- | --- |
|  | PHF5A expression | | P value | PHF5A expression | | P value |
|  | High | Low |  | High | Low |  |
| Gender |  |  |  |  |  |  |
| Male | 21 | 11 | 0.6937 | 32 | 22 | 0.6348 |
| Female | 17 | 11 |  | 8 | 4 |  |
| Age (years) |  |  |  |  |  |  |
| ≤60 | 16 | 5 | 0.1294 | 19 | 8 | 0.1768 |
| ＞60 | 22 | 17 |  | 21 | 18 |  |
| Pack-years |  |  |  |  |  |  |
| ≤30 | 30 | 19 | 0.4743 | 22 | 15 | 0.8295 |
| ＞30 | 8 | 3 |  | 18 | 11 |  |
| Tumor size |  |  |  |  |  |  |
| ≤5 | 30 | 15 | 0.3534 | 22 | 12 | 0.4823 |
| ＞5 | 8 | 7 |  | 18 | 14 |  |
| T status |  |  |  |  |  |  |
| T1+T2 | 26 | 14 | 0.7048 | 20 | 10 | 0.3576 |
| T3+T4 | 12 | 8 |  | 20 | 16 |  |
| N status |  |  |  |  |  |  |
| N0+N1 | 21 | 17 | 0.0882 | 33 | 21 | 0.8586 |
| N2+N3 | 17 | 5 |  | 7 | 5 |  |
| TNM stage |  |  |  |  |  |  |
| I+II | 17 | 16 | 0.0357 * | 23 | 14 | 0.7701 |
| III+IV | 21 | 6 |  | 17 | 12 |  |

* P < 0.05

**Supplementary Table S3. The PCR primers used to validate the AS of PHF5A-regulated genes.**

| Gene | Sense Primer | Antisense Primer |
| --- | --- | --- |
| ANAPC1 | GGAACTCTATGTTGCTGG | TAATACATGAGCTTTGCA |
| ANAPC10 | GACACCTCCTGGTGCTGA | TGTACGAGTTGGCTTCTT |
| ATR | TGCCGCTAATCTTCTAAC | GTGGCTTTCAAGTTCCTA |
| CHEK2 | TAAACGCCTGAAAGAAGC | GTCCCAACAGAAACAAGAAC |
| DBF4B | CCAGGACTGTGGAATCGG | GGTGAGTTCTTCTGGCATTTT |
| MLLT10 | AGCAAATCCTAGTCCGTCTC | TTGGTTACCTGGCAAAGC |
| SMC3 | GACTCGTGCCAAACTTGA | TTCACTATTGCCTGCTAG |
| SKP2 | AGCCCGACAGTGAGAACA | GAGACAGCAACCGACCAG |
| DNAJC19 | AGTTTGCGGCTGCTCCTG | AATGCTGCTTCCCGTTTT |
| MECP2 | AGGAGGAGGCGAGGAGGAGA | CCGGTCACGGATGATGGA |
| MITD1 | GGAAAATATCACAAGCAA | AATAATCAAGTCCCCTTC |
| MRPS12 | TTACGGAGGGACTTTCTGTTAG | TTGGCTGAGTTGGGCTTC |
| KDM6A | CTCTATGAATCCTGCAACC | ACAGCATGTCCACCACTC |
| ZNF585A | CCGCTGGTGTCAGGAGTA | GAGCAGGTGGCTGTAGGT |
| API5 | GACTGTGTGGACAGGCTCTT | TGCAAAGTACTGCAGCCTGA |
| BCL2L13 | AGAGAAGCTGCAAGAGCAACA | ACTCTGCCGAATAGTCCTCCA |
